# A Traumatic Impact Immediately Changes the Mechanical Properties of Articular Cartilage

**DOI:** 10.1177/19476035241235633

**Published:** 2024-03-19

**Authors:** Robin P. Blom, Danka Rahim, Erik Paardekam, Gino M. M. J. Kerkhoffs, Davide Iannuzzi, Theodoor H. Smit

**Affiliations:** 1 Department of Orthopedic Surgery and Sports Medicine, Amsterdam University Medical Centers, Amsterdam UMC location AMC, University of Amsterdam, Amsterdam, The Netherlands; 2 Amsterdam Movement Sciences, Sports, Amsterdam, The Netherlands; 3 Academic Center for Evidence-based Sports Medicine (ACES), Amsterdam, The Netherlands; 4 IOC Research Center, Amsterdam Collaboration on Health and Safety in Sports (ACHSS), Amsterdam, The Netherlands; 5 Department of Physics and Astronomy and LaserLaB, VU, Amsterdam, The Netherlands

**Keywords:** ankle, cartilage, impact, micro-indentation, osteoarthritis

## Abstract

**Objective:**

To investigate whether and how a single traumatic impact changes the mechanical properties of talar articular cartilage.

**Design:**

A marble was placed on the joint surface and a weight was dropped on both medial and lateral caprine talus to create a well-defined single focal impact. The mechanical properties of intact and impacted talar cartilage were measured with a micro-indenter. Elastic (storage) and viscous (loss) moduli were determined by oscillatory ramp and dynamic mechanical analysis protocols.

**Results:**

We found significant differences between ankles and within the same ankle joint, with the medial talus having significantly higher storage- and loss moduli than the lateral talus. The storage- and loss moduli of intact articular cartilage increased with greater indentation depths. However, postimpact the storage- and loss moduli were significantly and consistently lower in all specimens indicating immediate posttraumatic damage. The deeper regions of talar cartilage were less affected by the impact than the more superficial regions.

**Conclusions:**

A single traumatic impact results in an immediate and significant decrease of storage- and loss moduli. Further research must focus on the development of non- or minimally invasive diagnostic tools to address the exact microdamage caused by the impact. We speculate that the traumatic impact damaged the collagen fibers that confine the water-binding proteoglycans and thereby decreasing the hydrostatic pressure of cartilage. As part of the treatment directly after a trauma, one could imagine a reduction or restriction of peak loads to prevent the progression of the cascade towards PTOA of the ankle joint.

## Introduction

Osteoarthritis (OA) is the most common joint disorder^1^ and is considered a whole joint disease resulting in structural and finally functional failure of the joint.^2^ OA of the ankle is predominantly of posttraumatic origin^3,4^ and as a consequence, relatively young and active people have an increased risk of developing posttraumatic osteoarthritis (PTOA).^5^ PTOA of the ankle is caused by traumatic- or sports-related injuries ranging from a “simple” sprain to an intra-articular fracture.^6^ Because cartilage is not innervated and cartilage degradation itself is not painful, there is a long interval (up to decades) between the actual trauma and the first clinical symptoms of PTOA. This hinders early diagnosis directly after the trauma and subsequent intervention to prevent progression of the cascade toward PTOA.^7^

Despite ample clinical evidence that cartilage injuries initiate a slow process of progression toward PTOA, understanding of the etiology and pathogenesis is still limited.^8^ Cartilage is a highly organized tissue with chondrocytes embedded in the extracellular matrix (ECM). The ECM consists of type II collagen, proteoglycans, and water (with approximately 80% the predominant component). The proteoglycans are composed of sulfated glycosaminoglycans (sGAGs) side chains attached to hyaluronic acid backbones.^9^ The fixed negative charges of the sGAGs result in a high tonicity and influx of water into the cartilage. This induces tension on the collagen fibrils within the ECM and an increasing hydrostatic pressure, which is chondrogenic for chondrocytes that are the ECM producing cells within articular cartilage. This unique composition provides form and tensile strength, and determines the mechanical properties of articular cartilage: load distribution, stiffness to compression, and (together with synovial fluid) frictionless movement of articulating bones.^10^

It is well established that mechanical loading is a key determinant of cartilage structure and functioning.^11^ However, we believe that excessive articular loading (e.g., overloading with a single impact or a period of intensive physiological loading) is a key factor in the cascade of degeneration of articular cartilage toward PTOA.^11^ In our previous study, we found that immediately after a traumatic impact, cartilage is damaged, albeit not visible, even when evaluated on a micro-scale level by contrast-enhanced micro-computed tomography (CT).^12^

The primary objective of the current study is to investigate whether and how a traumatic impact changes the mechanical properties of talar articular cartilage in an *ex vivo* goat model using micro-indentation. The aim is to induce chondral microdamage and investigate the talar articular cartilage directly after the initial traumatic impact, because we believe that the collagen network will be disrupted. As a consequence of the traumatic impact, the proteoglycans flow out of the ECM, which in turn results in a loss of water and subsequently a decrease in hydrostatic pressure. Chondrocytes within the cartilage will then be subjected to distortional strain and stress, which induces a cascade resulting in catabolism and inflammation, which will lead to further distortion of the cartilage and finally PTOA. The flow of water in and out of the ECM and as a result in hydrostatic pressure, contributes toward the elastic modulus of articular cartilage. The mechanical properties of articular cartilage can be described by changes in its viscoelastic properties, with elasticity described by the storage modulus (a measure of how much energy must be put into the cartilage to distort it) and the viscosity described by the loss modulus (a measure of the energy dissipated or lost to synovial fluid flow).

The goal of the current study is to measure changes in the mechanical properties of talar articular cartilage directly after a single traumatic axial impact. Assessing early, subclinical cartilage damage directly after a trauma would raise the need for early intervention to prevent or restore minimal damage and thereby prevent progression of the cascade toward PTOA of the ankle.

## Methods

### Specimens

**Figure 1.**
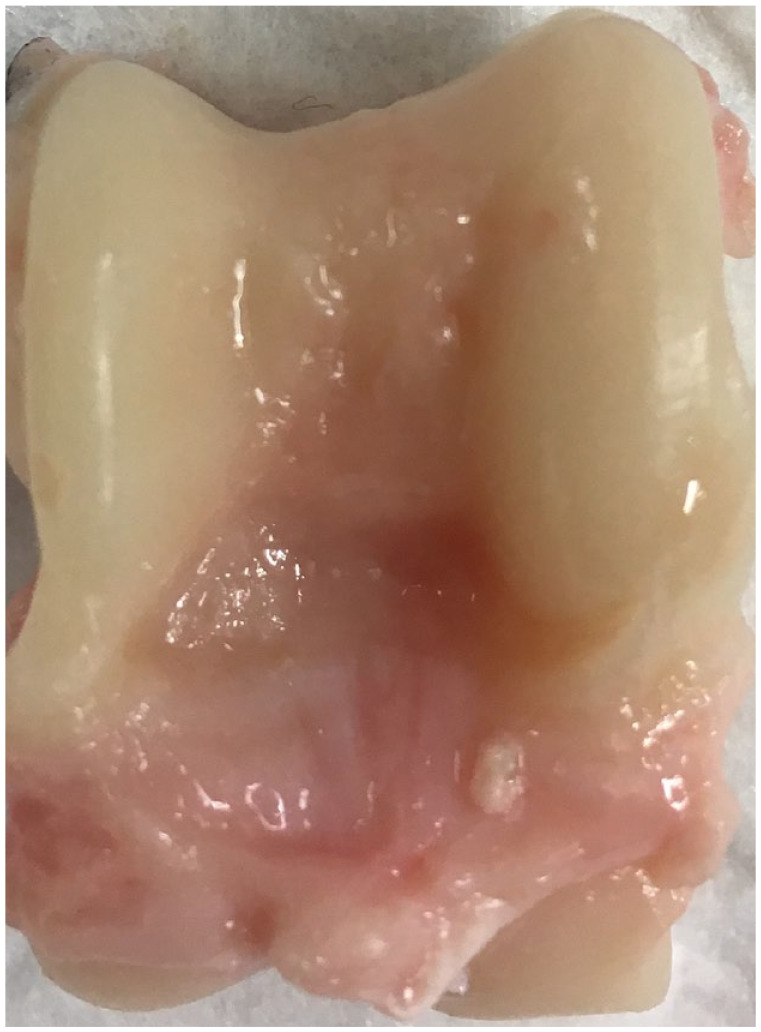
An example of a caprine talus and talar articular cartilage.

**Figure 2.**
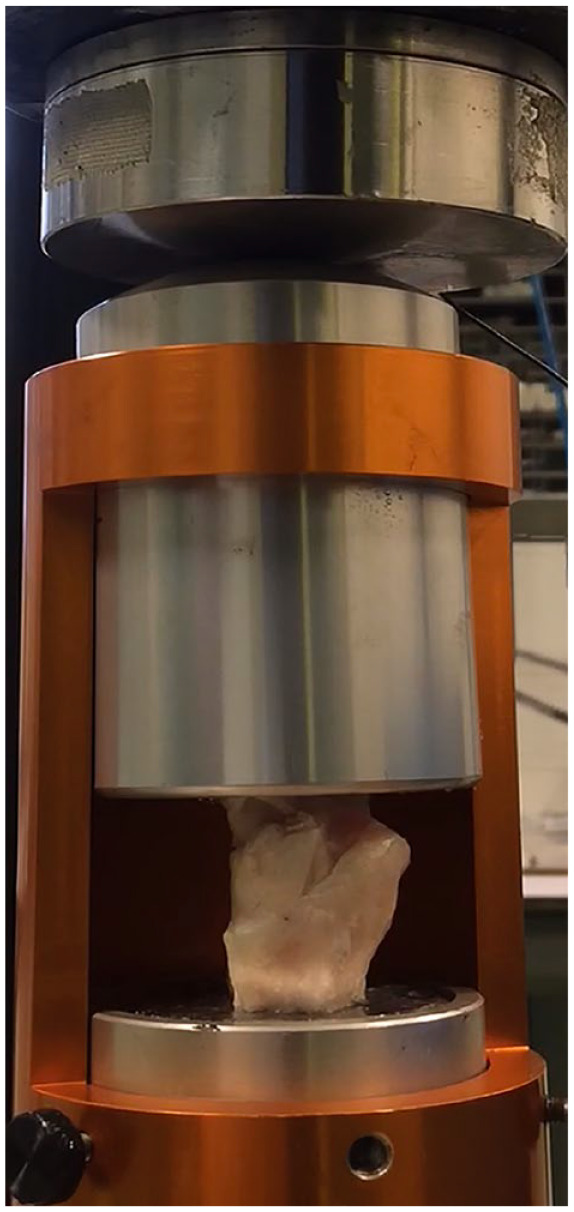
The impact testing set-up that allows for a controlled axial impact on the talus.

Sixteen fresh abattoir-derived (Firma Van der Horst, Maarssen, the Netherlands) Dutch milk goat (aged between 2 and 5 years) tarsocrural joints were used as an *ex vivo* joint model. Directly after harvesting the joints, all soft tissues were removed and the talus was dissected (Figure 1). The tarsal part of the talus was removed by transection of the talus, for the current study, we used that part of the talus that was previously part of the tibiotalar joint. Finally, the medial and lateral parts of the caprine talus were separated and directly immersed in phosphate-buffered saline (PBS) to prevent for dehydration. The distance of the cartilage regions of interest relative to the cutting (or transection) edges of the talar cartilage ranged from 5 to 10 mm. First, we investigated the mechanical properties of intact medial and lateral talar articular cartilage. Then, we applied a single impact, using a previously developed and described impact testing model^12^ that allows for a controlled axial impact on the talus (Figure 2). The talar specimens were exposed to a drop-weight of 2.5 kg released from a height of 10 cm. A marble with a diameter of 2 mm was placed between the tibiotalar joint surfaces to create a well-defined focal impact and create (superficial) chondral microdamage. This allowed for a direct comparison of each impacted region to its adjacent intact region for all samples. Caprine joints with impact induced macroscopic damage (e.g., intra-articular fractures) were excluded. Directly after impact testing, the samples were immersed in PBS to prevent for dehydration of the cartilage. Because of the preparations and the testing set-up, the time interval between the traumatic impact and the micro-indentation analysis was around 30 minutes.

### Micro-Indentation Technique

**Figure 3.**
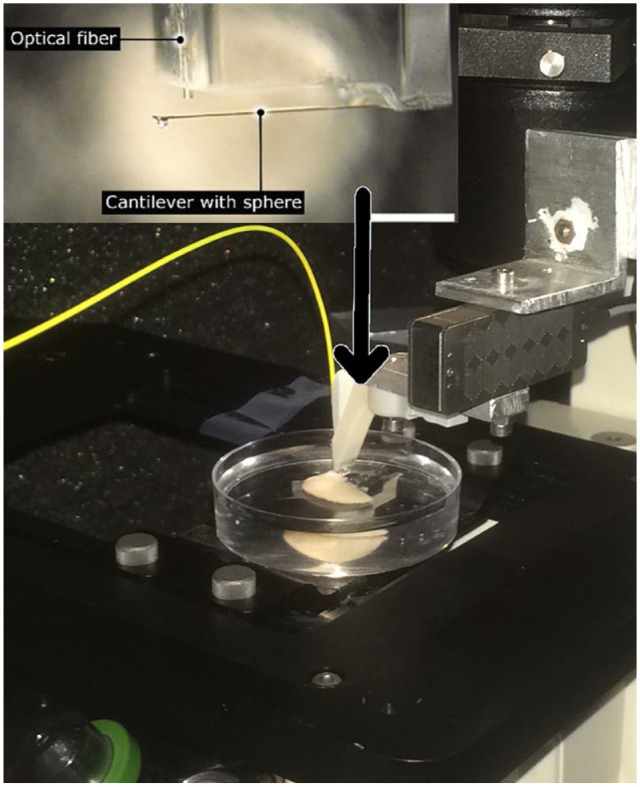
The micro-indentation set-up.

**Figure 4.**
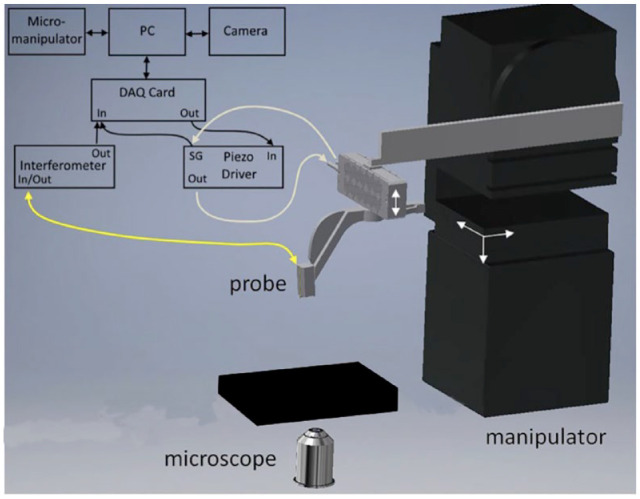
A schematic representation of the micro-indentation set-up.

A custom-made micro-indentation set-up was used in an acoustic isolation box.^13^ The indenter was custom-made and consisted of a ferrule-top probe with a microsphere on top of a cantilever spring^14^ to indent the talar articular cartilage^15^ (Figure 3). The spring constant of the cantilever was calibrated using precision digital weighing scale^16^ and the sphere size was measured with bright field microscope. The cantilever had a stiffness of k = 1,160 N/m and the ferrule-top probe a 250 µm bead radius to minimize the effect of local heterogeneities on a single measurement on the articular cartilage surface. The probe was equipped with an optical fiber to measure the cantilever deflection with the readout signal coupled to an interferometer (OP1550 developed and made by Optics11,^17^ Amsterdam, the Netherlands). The ferrule-top force transducer was mounted on a 3D printed holder, which was screwed to a PI 300 µm travel piezoelectric actuator (Physik Instrumente) and allowed for controlled movement during the actual indentation measurement. The piezoelectric actuator with the probe was mounted on a XYZ micromanipulator (Scientifica, PatchStar) to allow for longer-range movements (Figure 4). This set-up was able to apply an axial load and simultaneously calculate the indentation depth based on the measured cantilever deflection and extension of the piezoelectric actuator. Grids of measurements (5 × 2) with 250 µm spacing between the indentation locations were made to ensure that the indentation at each location is done on cartilage locations that have not previously been indented by the probe.

### Mechanical Properties of Articular Cartilage

Articular cartilage is described as a tissue with viscoelastic properties.^18^ The elastic response is described by the storage modulus; a measure of how much energy must be put into the cartilage to distort it. The viscous response is described by the loss modulus; a measure of the energy dissipated or lost to synovial fluid flow.

Unlike linearly elastic materials where all deformation energy is released during unloading, viscoelastic materials exhibit a damping effect, which causes a partial loss of energy due to the resistance against fluid flow. By a loading-unloading procedure during indentation we are able to calculate the changes in the viscoelastic responses. However, as cartilage is viscoelastic, this means a combination of ideal elastic response and ideal viscous response is more likely.

The method of data analysis and calculation of the storage and loss moduli was previously described by Antonovaite *et al.*^17^

### Oscillatory Ramp and Dynamic Mechanical Analysis Protocol

Two different indentation-controlled protocols^17^ were used to characterize the depth- and frequency-dependent viscoelasticity^19^ of talar articular cartilage. The oscillatory ramp loading protocol was used to determine how the viscoelastic response of talar articular cartilage changes with increasing indentation depth at a fixed oscillation frequency. In this protocol, the oscillation frequency is fixed and the indentation depth is simultaneously determined using closed-loop feedback. The current study used oscillations with amplitude of 0.1 µm and a frequency of 5.6 Hz.^17^ The indentation depth ranged from 1 up to 26 µm, this is the maximum indentation depth with using a 250 µm radius sphere while complying with one of the assumptions of the Hertz model.

Dynamic mechanical analysis (DMA)^20,21^ was the second indentation protocol and was designed to determine how the viscoelastic response of talar articular cartilage changes with ascending oscillation frequencies at a fixed equilibrium indentation depth of 24 µm.

In comparison with the oscillatory ramp loading protocol (fixed oscillation frequency and increasing indentation depth), the DMA protocol has an increasing oscillation frequency, but the indentation depth is fixed at an equilibrium indentation depth of 24 µm. Series of small sinusoidal oscillations at distinct frequencies, which are evenly spaced logarithmically between 1 and 10 Hz, respectively: 1.0, 1.8, 3.2, 5.6, and 10.0 Hz, were used. Data analyses were performed in MATLAB (The Mathworks Inc., Natick, MA) with custom-written functions.

### Statistical Analysis

The primary study outcome variables consisted of the storage and loss moduli of intact and impacted talar articular cartilage samples. Parametric data were presented as means and standard deviations. Non-parametric data were presented as medians and interquartile ranges. Normality of the data distribution was tested using the Shapiro-Wilk test. As parametric test, the paired *t* test was executed to compare the multiple groups, respectively, intact medial talar cartilage compared with intact lateral talar cartilage, intact medial talar cartilage compared with impacted medial talar cartilage and intact lateral talar cartilage compared with impacted lateral talar cartilage. For non-parametric primary outcome variables, the Wilcoxon signed-rank test was used to compare the multiple groups. A two-tailed Spearman correlation was used to determine whether a relationship existed between the (differences in) mechanical properties obtained from the micro-indentation tests and the different indentation depths and frequencies. The measured data were collected using MATLAB 2021a and statistical analysis was performed using SPSS Statistics 27. *P* < 0.05 was considered statistically significant.

## Results

This study found significant differences in mechanical properties between different talar articular cartilage specimens, however, there are also significant differences at different cartilage locations within the same talus.

### Medial Versus Lateral Intact Talar Articular Cartilage

**Figure 5.**
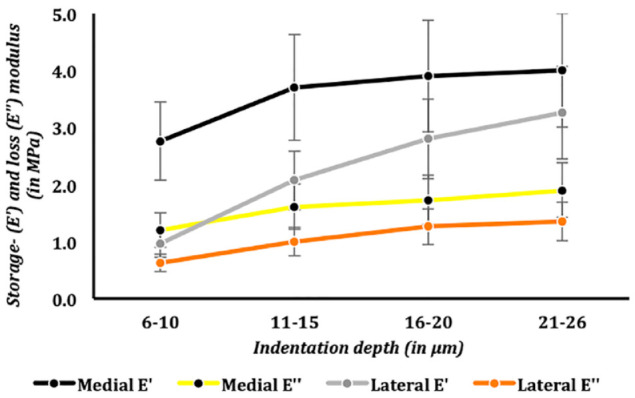
Storage and loss moduli in medial versus lateral intact talar articular cartilage, oscillatory ramp protocol.

**Figure 6.**
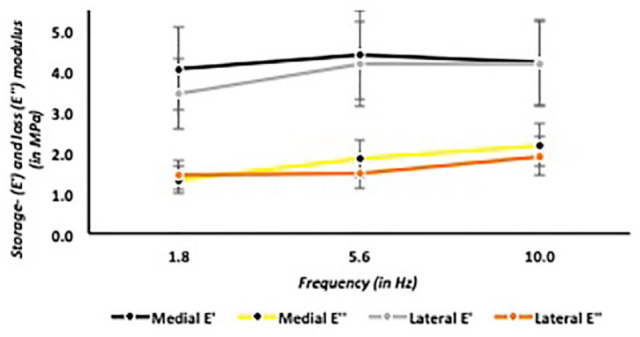
Storage and loss moduli in medial versus lateral intact talar articular cartilage, dynamic mechanical analysis (DMA) protocol.

Within the same caprine joints, the medial talus had significantly (*P* = 0.02) higher storage- and loss moduli compared with its adjacent lateral talus (Figure 5). However, at the relatively deeper indentation depths, ranging from 21 to 26 µm, there were no significant differences. These findings were confirmed using the DMA protocol, that is, there were no significant differences between medial and lateral talar articular cartilage at a fixed equilibrium indentation depth of 24 µm (Figure 6).

### Storage and Loss Moduli at Different Indentation Depths in Intact Talar Articular Cartilage

**Figure 7.**
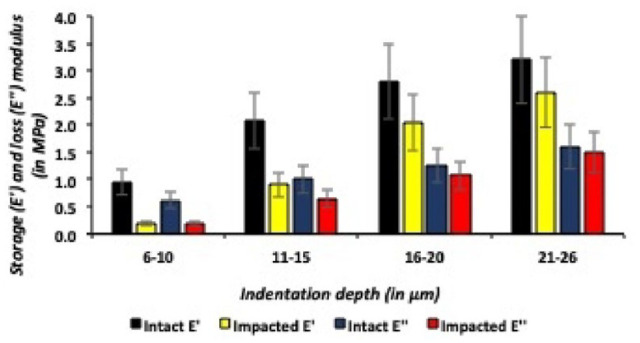
Storage and loss moduli (at different indentation depths) in intact and impacted lateral talar articular cartilage.

**Figure 8.**
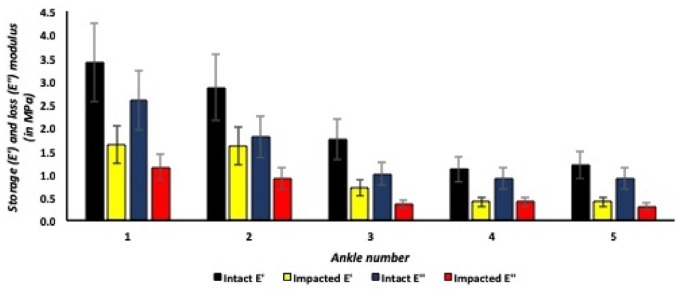
Storage and loss moduli in different lateral talar articular cartilage samples.

For both medial and lateral talar articular cartilage, the storage and loss moduli increased as the indentation depth increased in all talar specimens. For example, in the medial talus, when comparing the most superficial medial talar articular cartilage layers (6- 10 µm) to the relatively deeper layers (21-26 µm), the storage modulus (E’) significantly (*P* = 0.02) increased with 1.3 MPa. And for the lateral talus, when comparing the most superficial lateral talar articular cartilage layers (6-10 µm) to the relatively deeper layers (21-26 µm), resulted in a significant (*P* = 0.001) increase of the storage modulus (E’) of 2.0 MPa. This resulted in a significant positive correlation (Pearson’s r = 0.6) between the storage and loss moduli, and indentation depths were found in all caprine talar specimens. The most superficial talar articular cartilage layers, both medial and lateral talus, had significantly (*P* = 0.04) lower storage and loss moduli when compared with the relatively deeper talar articular cartilage layers at indentation depths between 21 and 26 µm (Figures 7 and 8).

### Intact Versus Impacted Medial Talar Articular Cartilage

**Figure 9.**
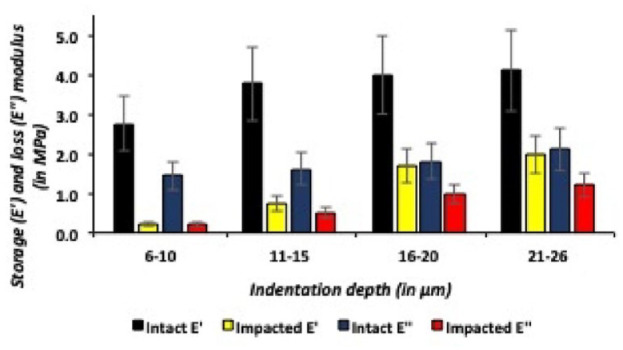
Storage (E’) and loss (E’’) moduli (at different indentation depths) in intact and impacted medial talar articular cartilage.

The traumatic impact resulted in significantly lower storage and loss moduli on the medial talus for all different indentation depths (6-10 µm; *P* = 0.001, 11-15 µm; *P* = 0.002, 16-20 µm; *P* = 0.02 and 21-26 µm; *P* = 0.03) when compared with its adjacent intact medial talar articular cartilage (Figure 9). As shown in Figure 7, when comparing intact with impacted medial talar articular cartilage, application of a single axial traumatic impact on the medial talus resulted in significantly lower E’ (storage moduli) and E’’ (loss moduli). The storage moduli (E’) medians for intact medial talar articular cartilage ranged from 2.8 to 4.1 MPa, and after application of a single axial impact significantly (*P* = 0.01) decreased to medians ranging from 0.2 to 2.0 MPa. The loss moduli (E”) for intact medial talar cartilage ranged from 1.4 to 2.1 MPa and after the application of a single axial impact significantly (*P* = 0.03) decreased with medians ranging from 0.2 to 1.2 MPa for impacted medial talar articular cartilage.

For medial talar articular cartilage, the intact:impact ratio of the storage modulus (E’) is 14.0 for the most superficial (6-10 µm) cartilage layers and significantly (*P* = 0.001) decreasing to 2.1 for the relatively deeper indentation depths (21-26 µm). And for the loss modulus (E”) of medial talar articular cartilage, the intact:impact ratio significantly (*P* = 0.02) decreased from on average of 7.0 in the most superficial (6-10 µm) medial cartilage layers to 1.75 at the relatively higher indentation depths (21-26 µm).

### Intact Versus Impacted Lateral Talar Articular Cartilage

**Figure 10.**
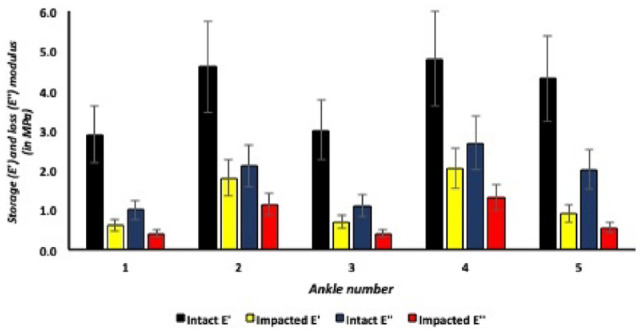
Storage and loss moduli in different medial talar articular cartilage samples.

**Figure 11.**
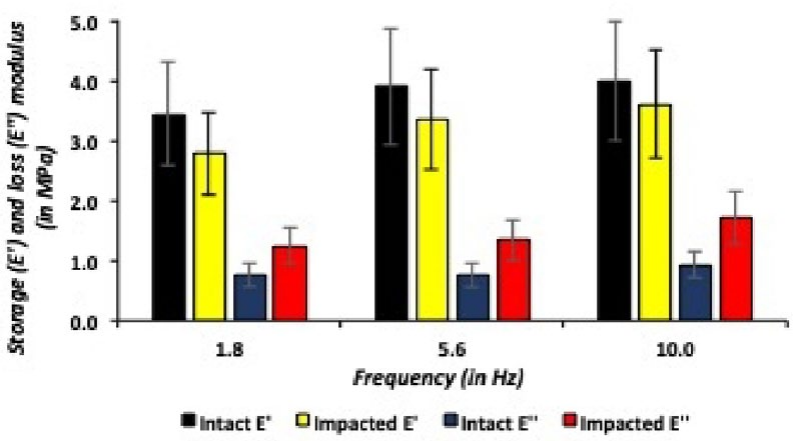
Storage and loss moduli in lateral talar articular cartilage, dynamic mechanical analysis (DMA) protocol.

Lateral talar articular cartilage showed lower storage (E’) and loss (E”) moduli after application of a single axial impact for all talar cartilage specimens (Figure 10). For example, the first ankle in Figure 10, the storage modulus (E’) for intact lateral talar articular cartilage is on average 3.5 MPa and significantly decreased (*P* = 0.01) after application of a single axial traumatic impact, resulting in a storage modulus (E’) of 1.7 MPa in impacted lateral talar articular cartilage. The same effect applies to the loss modulus (E”) with 2.6 MPa in intact lateral cartilage and a significant decrease (*P* = 0.03) to 1.3 MPa for axially impacted talar articular cartilage. The intact:impact ratio for the storage modulus (E’) is 5.0 for the most superficial lateral talar articular cartilage layers and decreasing to 1.2 for the relatively higher indentation depths in the lateral talus. The intact:impact ratio for the loss modulus (E”) decreased from on average 3.0 in the most superficial lateral talar articular cartilage layers (6-10 µm) to 1.1 in the relatively deeper cartilage layers (21-26 µm) of the lateral talus (Figure 11).

Figure 11 describes descriptive DMA data for lateral talar articular cartilage, revealing lower storage (E’) and loss moduli (E”) after application of a single axial traumatic impact on the lateral talar articular cartilage. Similar to the medial talus, all lateral talar articular cartilage specimens showed an increase in storage and loss moduli with ascending oscillating frequencies at the equilibrium indentation depth in the DMA protocol. In both the intact and impacted lateral talar articular cartilage specimens, the storage and loss moduli increased on average with a factor 1.3 as the indentation oscillating frequency increased within the DMA protocol.

## Discussion

PTOA is a slow process of articular cartilage degeneration. A trauma directly damages the articular cartilage and that effect should be measurable or detectable, although it appeared not visible, even when using contrast-enhanced micro-CT.^12^ The primary objective was to investigate whether and how a traumatic impact changes the mechanical properties of talar articular cartilage.

The current study revealed significant differences in mechanical properties between different talar articular cartilage specimens. However, we also showed the pre-existent differences within articular cartilage of the same caprine talus. Healthy and intact medial talar articular cartilage had significantly higher storage and loss moduli compared with intact lateral talar articular cartilage. This may be a regular mechanical adaptation to the activities of daily life and implies that one side of the talar joint cannot be used as control for the other side. Increasing storage and loss moduli at higher indentation depths were also observed in healthy or intact talar cartilage. As intact talar articular cartilage is not exposed to any impact, other factors, such as collagen fiber orientation or GAG variation, will differ per indentation depth. Previous research showed a linear relationship between the relative collagen and GAG contents versus depth or distance from the articular cartilage surface. The relative water content in articular cartilage is highest at the surface level and then decreases abruptly.^22^ The most superficial layer is in contact with synovial fluid and protects the deeper cartilage layers from shear stress due to articulation, where all layers but especially the relatively deeper cartilage layer provides resistance to compressive forces.^23^

Interestingly, the application of a single traumatic axial impact resulted in a significant decrease in both storage and loss moduli, which indicates direct posttraumatic damage to the talar articular cartilage. As previously described in the review by Kramer *et al.*,^24^ a traumatic impact results in the initiation of a few pathogenic processes by degradation of the ECM and finally PTOA. We hypothesized that a traumatic impact creates damage to the highly organized collagen network and as a consequence a loss of proteoglycans out of the ECM. This results in an efflux of water out of the ECM that decreases the hydrostatic pressure and changing the mechanical properties of caprine talar articular cartilage. As collagen fibers within the ECM can store energy elastically, it can be assumed that a traumatic impact could result in a damaged collagen network within the ECM. The decrease in hydrostatic pressure changes the loading mode of chondrocytes, from hydrostatic to shear stress. Chondrocytes within the ECM will become catabolic and induce inflammatory gene expressions that will initiate the degenerative cascade toward PTOA of the ankle cartilage. The observed decline in both storage (E’) and loss moduli (E”) of impacted cartilage implies that the ability of cartilage to store energy for elastic recoil and the ability to dissipate energy have decreased.

As expected, the relatively deeper articular cartilage regions (21-26 µm) of caprine talus were less affected by the traumatic impact compared with the more superficial layers (6-10 µm). Superficially, the collagen fibers are packed tightly and aligned parallel to the articular surface. Due to this organization and orientation of the collagen network, it is more capable to absorb shear stress instead of the axial stress as we applied with a single traumatic axial impact to the talar articular cartilage.

The current study findings should be interpreted in the light of its strengths and limitations. A limitation is the use of caprine cartilage instead of human talar articular cartilage samples. For this reason, we have to be careful to extrapolate the current study findings to human talar articular cartilage. As human bodies donated for medical scientific research purposes are often of an older age, with a higher prevalence of OA, they were not suited for the current study aim. Caprine and human cartilage are both comparable in terms of viscoelasticity properties, but caprine cartilage is somewhat stiffer than human articular cartilage.^25-27^ Another difference is the thickness of articular cartilage; previously published studies described thicknesses between 1.0 and 1.5 mm on average for the human talus and between 0.3 and 0.6 mm on average for the caprine talus.^28-30^ However, as we aimed to induce focal chondral damage or articular cartilage defect on micro-scale level, we analyzed the effect of a single axial traumatic impact within the same talar cartilage specimens. Because of the previously described variations between different animals and joints,^30,31^ we are not able to compare between different talar articular cartilage specimens. The goal was to create an analogous situation to the human talus (for example, peak pressure or high compressive loads in the talus, during or directly after an inversion trauma of the ankle joint) using a reproducible and specifically well-defined impact in a caprine joint model.

Interestingly, the effect of the traumatic impact was significantly higher in the superficial layers of talar articular cartilage, especially in the medial talus. This significant difference in effect of traumatic impact between the medial and lateral talus is interesting from a clinical perspective, as talar osteochondral defects (OCDs) are often located at the superomedial corner of the human talus.^32^ It is generally accepted that talar OCDs are the result of an inversion trauma with stretching or disruption of the lateral ligamentous complex of the ankle joint and an impact or concussion of the medial talar articular cartilage.

In summary, a single traumatic axial impact directly changes the mechanical properties of talar articular cartilage, albeit not visible, even when evaluated on a micro-scale level by contrast-enhanced micro-CT. Further research should focus on the exact microdamage caused by the impact, we speculate that the traumatic axial impact damages the collagen network that confine the water-binding proteoglycans in the ECM and thereby release the pre-stress of the collagen fibers. This would change the loading mode of the chondrocytes from hydrostatic to shear stress and thereby inducing a cascade of catabolic and inflammatory processes toward PTOA of the ankle joint.

Articular cartilage damage directly after a traumatic impact is inevitable, but we need earlier and better detection of microdamage of the articular cartilage surface. Future research must focus on the development of diagnostic tools, for example, the clinical application of micro-indentation during minimally invasive ankle arthroscopy. Furthermore, as part of the earlier and better treatment of damaged articular cartilage directly after a trauma, one could imagine a reduction or restriction of peak loads. This could prevent the progression into the next step in the cascade^33^ or vicious circle^34^ toward PTOA of the ankle joint.
